# Transpubic Urethroplasty: A Single Center Experience

**DOI:** 10.1155/2014/826710

**Published:** 2014-06-09

**Authors:** Raj Kumar Mathur, Niraj Shriram Tiwari, Sudharshan A. Odiya

**Affiliations:** Department of Surgery, MGM Medical College & MY Hospital, Indore 452001, India

## Abstract

*Objective.* To evaluate the long-term results of transpubic urethroplasty for pelvic fracture urethral distraction defects. *Patients and Methods.* Sixteen patients who had undergone transpubic urethroplasty for posttraumatic complex posterior urethral disruptions between 2007 and 2013 were analyzed retrospectively and prospectively. Patients were followed up for a mean (range) of 24 (6–60) months by history, urinary flow rate estimate, retrograde urethrography, and voiding cystourethrography. *Results*. The mean age of the patients was 30.4 years. The estimated radiographic stricture length before surgery was 4.3 cm. Transpubic urethroplasty was successful in 14 out of 16 patients. Postoperative complications were recurrent stricture (12.5%), urethrocutaneous fistula (12.5%), incontinence (31.25%), impotence (25%), and wound infection (18.75%). Failed repairs were successfully managed endoscopically in one patient and by perineal anastomotic repair in the other, giving a final success rate of 100%. Five out of 16 patients were incontinent of which 3 of them resolved and 2 had permanent incontinence. Impotence was seen in 4 out of 16 patients. There were no reported complications of pubectomy in any of our patients. *Conclusions.* Though considered obsolete now, transpubic urethroplasty for complex posterior urethral disruptions is still a viable alternative with excellent results and minimal morbidity.

## 1. Introduction

The outlook for the patient with a pelvic fracture urethral distraction defects has improved almost beyond recognition in the last decade as a result of the development of variety of anastomotic techniques and novel approaches for urethral reconstruction. The combination of the relatively restricted surgical access together with its inherent sphincter function makes any reconstruction of the posterior urethra a much more complicated procedure [1, 2].

Complex posterior urethral disruptions continue to represent a genuine challenge and they pose one of the most difficult management problems in urology, which represents 5% of all pelvic fracture urethral distraction defects. It is characterized by a stricture gap exceeding 3 cm, previous failed repair, associated perineal fistulas, rectourethral fistulas, periurethral cavities, false passages, or an open bladder neck [3, 4].

Although the published work is abundant with guidelines for reconstruction of the more common pelvic fracture-associated urethral disruptions, management algorithms for complex urethral injuries are scant [5, 6].

Over a 6-year period, we have managed sixteen cases of traumatic complex posterior urethral disruptions with a preliminary suprapubic cystostomy and delayed urethroplasty by a transpubic approach.

## 2. Materials and Methods

The surgical records at our institute were reviewed to identify patients who had had a transpubic urethroplasty for posttraumatic complex posterior urethral disruption between 2007 and 2013. In all, 16 patients, with a mean (SD, range) age of 30.4 (14–42) years, were identified. Their hospital and office charts were reviewed, noting patient age, urinary retention at the time of presentation, cause of stricture, treatment complications, and the duration of catheter drainage after surgery.

In all patients, the evaluation before surgery included a history, physical examination, retrograde, and voiding cystourethrography. Intravenous antibiotics were administered perioperatively and then tailored according to urine sensitivity. Urethroplasty was performed only if the urine culture was sterile.

A midline perineal exposure of the distal site of stricture was made. The normal distal urethra was circumferentially mobilized (circumurethral mobilization) and detached for a few centimeters from the crura. Distal corporeal body separation for a similar length was achieved (corporal separation). The distal urethral tube was trimmed back to freshly bleeding edges. A vertical midline infraumbilical incision was made up to the base of the penis. The recti were split to enter the prevesical space. The suspensory ligament of the penis was divided, the penis was retracted inferiorly, and the pubic symphysis was bared of muscular attachments. A pubectomy provided adequate exposure of the posterior urethral disruption. Adjacent fibrous tissue, callus, fracture fragments, and false passages were excised. After satisfactory mobilization of the proximal and distal urethra, the proximal urethra was tacked to the prostatic tissue with absorbable, interrupted sutures. A spatulated elliptical end-to-end anastomosis over a 16-18F silicon Foley's catheter was performed. After placing a suprapubic cystostomy, the incisions were closed.

Urethral stenting with silicon catheter and suprapubic cystostomy drainage were maintained for 21–28 days after surgery. After removing the urethral catheter, a gravity voiding film, with contrast medium infused through the suprapubic cystostomy tube, was taken to confirm the integrity of the repair, and if found satisfactory, the suprapubic cystostomy catheter was removed 1 day later. Patients were followed at regular intervals and evaluated symptomatically by history. Urinary flow studies were used routinely.

On suspicion of a recurrent stricture (recurrent obstructive symptoms, poor flow rates, or large postvoid residual urine), a retrograde urethrogram was taken. The eventual surgical success was defined as asymptomatic voiding with no clinical evidence of residual stricture (good flow rate and no residual urine).

## 3. Results

All patients had urethral disruptions associated with trauma, caused by a motor vehicle in 13 (82%) and a fall from height in 3 (18%); the trauma was associated with a pelvic fracture in all patients with Tile's type B being the most common. The classical presentation in all patients was with a suprapubic cystostomy tube and scheduled for delayed or repeated correction of a urethral disruption. Six patients (38%) were referred to us after previous attempts at repair, including repeated endoscopic internal urethrotomy in one and failed attempts at open perineal reconstruction of posterior urethral distraction defects in five. The interval between the original trauma and repair in new cases and since the last repair in recurrent cases was 3–24 months.

The stricture length and location were analyzed by reviewing urethrography and operative details; the mean (range) length was 4.3 (3.4–5.5) cm with bulbomembranous being the most common site in our series (Table 1). All patients underwent transpubic urethroplasty with no early complications of surgery. The mean (range) follow-up was 24 (6–60) months. In two of our patients, the disease recurred and open surgical revision (perineal approach) was required in one, and the other was managed by visual internal urethrotomy. In none of the other patients was a secondary procedure required. All recurrent strictures were short, at the site of the anastomosis, and within the first year after surgery. Thus, the primary success rate was 87.5% and the overall success rate was 100%.

Incontinence (Table 2) was seen in 5 out of 16 patients (31.25%) of which 3 improved within 6 months of surgery with perineal exercises and the other 2 remained incontinent till follow-up. Impotency was observed in 4 of our patients (25%) initially with some improvement seen in two of them on follow-up visits. Two (12.5%) patients developed urethrocutaneous fistulae which were treated conservatively and fistulae closed without intervention. Superficial wound infection of perineal wound occurred in 3 (18.75%) cases. It was a minor infection and was managed with drainage of pus, local antiseptic dressings, and antibiotics.

## 4. Discussion

Pelvic fracture urethral distraction defects (PFUDD) represent an uncommon but a difficult urological problem. Such urethral disruptions generally have an acquired cause, as congenital and infectious strictures are rare. The choice of management has varied from primary urethral realignment/anastomosis to cystostomy and delayed urethroplasty. The management of associated nonurological injuries takes precedence to attain haemodynamic stability, and suprapubic diversion controls the urological injury [7].

Surgical intervention for complex posterior urethral disruption is intricate because of limited urethral length, surrounding fibrosis, and distorted anatomy of the pelvis. Such types of strictures have been dealt with by perineal elaboration technique or abdominal transpubic perineal technique [3, 8, 9] but the standard approach to such patients has always been elusive to the urologists. Many urologists still prefer perineal approach for posterior urethral disruption but this approach has its limitations and may not adhere to the principles of widely spatulated tension free anastomosis.

Total removal of the symphysis pubis, first reported by Pierce in 1962, has been recommended when severe injuries result in complicating features such as fistula or marked displacement or retropubic fixation of the prostate. Careful planning and meticulous operative techniques using transpubic approach can help us achieve satisfactory results even in such complex cases. The keys to a successful repair in these patients are adequate exposure of the pelvic anatomy, extraperitoneal approach, simultaneous bladder neck reconstruction, protecting the urethral anastomosis, and obliterating pelvic dead space.

Urinary incontinence and erectile dysfunction are the main concern for patients having complex posterior urethral disruptions. In our series, 5 out of 16 (31.25%) patients were incontinent, three (18.75%) of them improved with perineal exercises while the other two (12.5%) remained incontinent till follow-up. This can be attributed to a host of factors including anatomic level of urethral disruption, associated pelvic fractures, management of primary injury, dissection around the bladder neck, and proximal urethra at urethroplasty, retropubic infections, and reoperations [10, 11]. We believe that the best management of incontinence lies in its prevention by meticulous dissection techniques and thorough knowledge of anatomy.

In our series, impotence was seen in 4 out of 16 (25%) patients, 3 (18.75%) of them prior to and 1 (6.25%) after surgery. Preservation of potency was vital but could not be achieved in all cases due to extensive retropubic dissection required in some cases.

None of the patients surgically treated had any complications related to pubectomy such as defect of posture or gait.

## 5. Conclusion

Althoughthe reference standard for treating pelvic fracture urethral distraction defects is transperineal one-stage bulboprostatic anastomosis, transpubic urethroplasty has opened new horizons for a urologist to achieve a tension- and scar-free bulboprostatic urethral anastomosis with minimal postoperative complications for what was first described as a “*hopeless urethra.*” We are looking forward to further minimizing the morbidity associated with this procedure as it has a steep learning curve and requires much more expertise than other fellow procedures.

## Figures and Tables

**Table 1:** Site of strictures.

Site	Number of patients (out of 16)	Percentage
Membranous + Bulbar	11	68.75%
Prostatic + Membranous + Bulbar	2	12.5%
Prostatic + Membranous	3	18.75%

**Table 2:** Complication rates.

Complication	Frequency	Percentage
Infection	3	18.75%
Restenosis	2	12.5%
Incontinence	5	31.25%
Impotence	4	25%
Urethra cutaneous fistula	2	12.5%
Pubectomy related	—	—
